# Pilot and feasibility studies come of age!

**DOI:** 10.1186/2055-5784-1-1

**Published:** 2015-01-12

**Authors:** Gillian A Lancaster

**Affiliations:** Postgraduate Statistics Centre, Department of Mathematics and Statistics, Lancaster University, Lancaster, LA1 4YF UK

## Abstract

This editorial introduces the new, online, open-access journal *Pilot and Feasibility Studies*. The journal considers manuscripts on any aspect of the design and analysis of pilot and feasibility studies, as well as protocols for pilot and feasibility studies, and discussions and reviews of methodological issues around the planning and reporting of such studies. These studies are generally carried out in preparation for future large-scale definitive randomised controlled trials or observational studies and address key issues of uncertainty. Objectives for conducting pilot and feasibility studies therefore differ from those of the future large-scale study and should be clearly expressed. The journal provides a dedicated place for publication of this important work as well as a forum for discussion of methodological issues that will lead to increased scientific rigour in this area.

## Introduction

Welcome to this first edition of a new journal called *Pilot and Feasibility Studies*. This is an exciting addition to the BioMed Central portfolio of journals and is long overdue. The journal is online and open-access ensuring that it is available to researchers from all over the world. Moreover, because of its multi-disciplinary nature, researchers will benefit from seeing a range of pilot and feasibility studies reported from across many subject areas, facilitating the sharing of ideas. There will be a strong focus on pilot and feasibility studies that are in preparation for a randomised controlled trial, the gold standard of study designs, but this is by no means exclusive to other types of studies, and certainly similar preparation should take place for observational study designs, too. The key issue here is uncertainty and how that is to be addressed when focusing upon planning a future large-scale study.

Previously, many pilot and feasibility studies have remained unpublished, and yet this work is crucial to the success of a future trial. It is encouraging that pilot trials are now being registered within National Trials registries to improve transparency and accountability, not least to funders [1]. Trial registration is also a condition for publication in the BioMed Central sister journal *Trials* [2]. There is however still a way to go in the reporting of other types of pilot and feasibility studies, although more are being registered on the UK Clinical Research Network Portfolio Database (http://public.ukcrn.org.uk). These are of equal importance in ensuring that all processes work and run smoothly, that interventions are safe and have shown efficacy, and may include qualitative work that helps us to more easily understand and appreciate patients’ and health professionals’ perspectives [3], and cost effectiveness modelling [4]. *Pilot and Feasibility Studies* provides a ready forum for discussion around these key aspects of the scientific process, sharing knowledge in the planning of large-scale clinical investigations and so completing the publication cycle for clinical and health research.

The aim of this journal is to provide a dedicated place for the reporting of feasibility and pilot studies, and discussion of methodological issues around the planning, of future large-scale definitive trials and observational studies. Articles may include randomised pilot trials, non-randomised feasibility and pilot studies, methodological discussion and review papers, protocols for pilot and feasibility studies, qualitative work in preparation for a large-scale study, phase II studies that address preparatory work for a future trial, proof-of-concept studies, Vanguard studies and similar related studies not covered within the above terminology. The main focus of the above is on external stand-alone pilot and feasibility studies. However, with more attention being placed on adaptive trial designs and internal pilot studies, then submissions on internal pilot studies that address interesting preparatory work and/or innovative adaption are also encouraged.

### Guidelines for the conduct and reporting of pilot and feasibility studies

The Medical Research Council (MRC) and National Institute for Health Research (NIHR) have both published guidelines for conducting pilot and feasibility studies. The MRC guidelines on complex interventions present the view that pilot and feasibility testing are interchangeable concepts covering all aspects of preparatory work [5] (see http://www.mrc.ac.uk/complexinterventionsguidance):

‘*Questions to ask yourself include: Have you done enough piloting and feasibility work to be confident that the intervention can be delivered as intended? Can you make safe assumptions about effect sizes and variability, and rates of recruitment and retention in the main evaluation study? What design are you going to use, and why? Is an experimental design preferable and if so, is it feasible?*’

In the NIHR online glossary feasibility studies and pilot studies are described in two separate entries. There are examples of feasibility parameters that may need to be tested in preparation for the main trial, and there is a suggestion that this process might come before more substantial pilot work (see ‘Feasibility studies’ and ‘Pilot studies’ entries at http://www.nets.nihr.ac.uk/glossary):

‘Pilot studies are a smaller version of the main study used to test whether the components of the main study can all work together. It is focused on the processes of the main study, for example to ensure that recruitment, randomisation, treatment, and follow-up assessments all run smoothly.’

Useful guidelines for conducting pilot and feasibility studies have also been published in other subject-specific areas, including occupational therapy [6], critical care [7], perinatal and neonatal nursing [8], nursing science [9], psychiatry [10] and social research [11]. However, there exists little guidance on how to report pilot and feasibility studies.

In 2012, a consortium of six people (see the ‘Acknowledgements’ section), including myself, set out to develop new Consolidated Standards of Reporting Trials (CONSORT) extension guidelines for reporting pilot and feasibility studies. The original CONSORT guidelines were most recently updated in 2010 and focus on the reporting of randomised controlled trials [12]. Extensions to these guidelines, for example, to cluster randomised trials [13] and pragmatic trials [14] have since been developed. We had previously published articles on the conduct of pilot and feasibility studies and presented conference workshops on the subject, which highlighted a clear need for such guidelines. Since then, a comprehensive body of work has been carried out, which has included consulting experts in the field, journal editors and funders. Our work has resulted in a set of reporting guidelines for pilot trials, which is currently being written up, and in fact the creation of this journal is a very positive indication of publisher support for this work.

As well as developing the new CONSORT extension guidelines for pilot and feasibility trials, consideration has also been given to the construction of definitions as to what constitutes a ‘feasibility study’ and ‘pilot study’, which has given rise to much interesting debate. To date, our work has shown that there is a lack of consensus over the different usage of the words ‘pilot’ and ‘feasibility’ in this context. Consequently, both terms are currently being used interchangeably in this journal. Our work is soon to be published and this will hopefully open up new avenues of debate around terminology.

### Key objectives of pilot and feasibility studies

A major issue of concern in previously published work has been around appropriate objectives for conducting a pilot or feasibility study. These objectives should be different from those of the future definitive study and should stipulate the issues of uncertainty to be addressed in preparation for the future large-scale study. Moreover, pilot and feasibility studies are not designed (or powered) to address the effectiveness of the intervention. This is an objective of the future definitive trial. Nevertheless, these are issues that often arise in publications and lead to confusion of the reasons for a pilot or feasibility study.

In 2004, Lancaster et al. [15] published recommendations for good practice in relation to the design of pilot and feasibility studies, and highlighted seven evidence-based key objectives found in their literature review of pilot and feasibility papers published in 2000/2001. These are (i) to test the integrity of the study protocol for the future trial (which gives a valid reason for randomisation) and (ii) to gain initial estimates for sample size calculation, and it is helpful to note here recent work in this area [16, 17] (iii) to test data collection forms or questionnaires, (iv) to test randomisation procedure(s), (v) to estimate rates of recruitment and consent, (vi) to determine the acceptability of the intervention and (vii) to select the most appropriate primary outcome measure(s). This paper was written to encourage researchers to carry out pilot and feasibility studies for the right reasons and to deter them from conducting mini-randomised controlled trials that mirrored the main trial in all respects, except that they were carried out on smaller numbers of participants. One reason for this might be, for example, because it was easier to conduct the study in a single centre rather than carrying it out more appropriately across multiple centres to increase sample size.

Further work published in 2010 in relation to cluster randomised trials and complex interventions, resulting from talks given at the Royal Statistical Society Primary Health Care Study Group meetings over a 10-year period, highlights other key objectives [18]. In the same year, Thabane et al. published a tutorial on pilot studies [19]. They also agreed that there were often no clear criteria regarding feasibility objectives in proposals seen in their work with research ethics boards. They further recommended when possible formulating *a priori* ‘criteria for success’ in interpreting the results of pilot and feasibility studies, for example, percentage achievement of recruitment targets or acceptability objectives, and put forward a checklist of items for reporting results based on CONSORT.

A second related issue is the appropriate statistical analysis of pilot and feasibility studies, and whether the use of hypothesis tests is acceptable in small under-powered studies. McGrath [8] puts it this way:

‘*Pilot studies because they are often underpowered (small sample sizes) can only be used to generate beliefs that there will be a trend toward significance that provide support for larger studies. If significance is found in a pilot study, one must still be prepared that a full-scale trial could generate different results*.’

Lancaster et al. [15] called for more emphasis to be placed on confidence interval estimation rather than hypothesis testing as then the imprecision of the estimates can be clearly seen. Arain et al. [20] repeated the search carried out by Lancaster et al. in 2001/2002 [15] for the years 2007/2008 to see if current practice had changed. They found that the majority (81%) of the 54 pilot and feasibility studies identified from their search still incorporated hypothesis testing and some included tests of treatment effectiveness. Nevertheless, this is evidence that many journals continue to condone hypothesis testing in small pilot or feasibility studies. Given that this practice is unlikely to change in the near future, then we would stipulate that the emphasis given to hypothesis testing should be treated as secondary and the results treated with caution. This is in line with the recommendations of Thabane et al. [19].

Whether or not hypothesis testing is to be used, what is clear is that the main objective of a pilot or feasibility study is not to test treatment effectiveness [10, 15, 19]. This is the objective of the future definitive trial. Therefore, any preliminary testing should be clearly labelled as such and issues of concern pointed out in the discussion section of a paper. However, it is understood that there may be instances when the collection of patient-related outcome data might serve a useful purpose in future planning. However, this would not generally be the primary focus of a pilot or feasibility study, as the main objective(s) should be concerned with addressing aspects of feasibility and uncertainty.

A third issue related to the reporting of pilot and feasibility studies is that it is important to state in the final conclusions whether the aims and objectives of the pilot or feasibility work have been met and are going to lead on to a future large-scale study. In a recent review by Shanyinde et al. [21], coverage of feasibility issues in the discussion section of a paper was classified as ‘none’ or ‘minimal’ in 17/28 drug-related pilot trials and in 5/22 non-drug pilot trials, and the extent of discussion about planning future trials was classified as ‘none’ or ‘minimal’ in 27/28 of the pilot drug trials and 14/22 non-drug trials. This highlights that this important issue is not given enough consideration. In many cases, the intension for further work is left far too vague and this has been illustrated by other authors [15, 19, 20]. Bugge et al. [22] have put forward a process for decision-making after pilot and feasibility trials (ADePT). It can be used to classify and analyse problems arising from the feasibility study as an aid to identifying appropriate solutions, and they provide a detailed example related to pelvic organ prolapse.

Several authors also warn against the pitfalls of including the same pilot study participants in the main study when external stand-alone pilot studies are carried out. They highlight in this respect that the main study may suffer from contamination of the study sample because of modification to study methods after the pilot study has completed [10], or because of previous exposure to the intervention, or because of selection bias caused by unrepresentative pilot sampling [11], for example, where participants are recruited opportunistically and may not truly reflect the target population of interest in the future large-scale study. Charlesworth et al. [23] have proposed a checklist for deciding whether pilot trial data can be carried forward to the main trial without compromising trial integrity, including consideration of acceptable permissible changes to procedures between the pilot and main trial. This may also prove helpful for use with adaptive trial designs.

## Conclusion

In conclusion, piloting new interventions for use in definitive randomised controlled trials or testing the feasibility of other aspects of uncertainty arising in the development of large-scale studies ensures that the methodological approach taken in the main study is robust and feasible. These are important parts of the development process and provide evidence to funders that the future main trial will work. Pilot and feasibility studies encompass all aspects of the design process, and whilst this work is crucial to the success of a future trial, in the past, such studies have seldom reached publication for a variety of reasons. Having an open-access journal dedicated to supporting this type of work is long overdue. It will enable researchers to learn from each other, discuss and review previous approaches and share more easily ideas for best scientific practice across subject areas.

We invite you to submit your papers to *Pilot and Feasibility Studies* and to contribute to a new era in the publication of such work, as pilot and feasibility studies come of age!
